# Efficient lactic acid production from dilute acid-pretreated lignocellulosic biomass by a synthetic consortium of engineered *Pseudomonas putida* and *Bacillus coagulans*

**DOI:** 10.1186/s13068-021-02078-7

**Published:** 2021-11-27

**Authors:** Lihua Zou, Shuiping Ouyang, Yueli Hu, Zhaojuan Zheng, Jia Ouyang

**Affiliations:** 1 Jiangsu Co-Innovation Center of Efficient Processing and Utilization of Forest Resources, College of Chemical Engineering, Nanjing Forestry University, Nanjing, 210037 People’s Republic of China; 2 Jiangsu Province Key Laboratory of Green Biomass-Based Fuels and Chemicals, Nanjing, 210037 People’s Republic of China

**Keywords:** Lignocellulosic hydrolysate, Detoxification, *Pseudomonas putida*, *Bacillus coagulans*, Microbial consortium, Lactic acid

## Abstract

**Background:**

Lignocellulosic biomass is an attractive and sustainable alternative to petroleum-based feedstock for the production of a range of biochemicals, and pretreatment is generally regarded as indispensable for its biorefinery. However, various inhibitors that severely hinder the growth and fermentation of microorganisms are inevitably produced during the pretreatment of lignocellulose. Presently, there are few reports on a single microorganism that can detoxify or tolerate toxic mixtures of pretreated lignocellulose hydrolysate while effectively transforming sugar components into valuable compounds. Alternatively, microbial coculture provides a simpler and more efficacious way to realize this goal by distributing metabolic functions among different specialized strains.

**Results:**

In this study, a novel synthetic microbial consortium, which is composed of a responsible for detoxification bacterium engineered *Pseudomonas putida* KT2440 and a lactic acid production specialist *Bacillus coagulans* NL01, was developed to directly produce lactic acid from highly toxic lignocellulosic hydrolysate. The engineered *P. putida* with deletion of the sugar metabolism pathway was unable to consume the major fermentable sugars of lignocellulosic hydrolysate but exhibited great tolerance to 10 g/L sodium acetate, 5 g/L levulinic acid, 10 mM furfural and HMF as well as 2 g/L monophenol compound. In addition, the engineered strain rapidly removed diverse inhibitors of real hydrolysate. The degradation rate of organic acids (acetate, levulinic acid) and the conversion rate of furan aldehyde were both 100%, and the removal rate of most monoaromatic compounds remained at approximately 90%. With detoxification using engineered *P. putida* for 24 h, the 30% (v/v) hydrolysate was fermented to 35.8 g/L lactic acid by *B. coagulans* with a lactic acid yield of 0.8 g/g total sugars. Compared with that of the single culture of *B. coagulans* without lactic acid production, the fermentation performance of microbial coculture was significantly improved.

**Conclusions:**

The microbial coculture system constructed in this study demonstrated the strong potential of the process for the biosynthesis of valuable products from lignocellulosic hydrolysates containing high concentrations of complex inhibitors by specifically recruiting consortia of robust microorganisms with desirable characteristics and also provided a feasible and attractive method for the bioconversion of lignocellulosic biomass to other value-added biochemicals.

**Supplementary Information:**

The online version contains supplementary material available at 10.1186/s13068-021-02078-7.

## Background

Biochemicals produced from renewable resources through microbial fermentation are rapidly garnering interest due to the exhaustion of fossil fuels and associated environmental issues [1]. Among these biochemicals, lactic acid and its derivatives are increasingly important building blocks, which have broad applications in the food and chemical industries [2]. Currently, the biological approach for lactic acid production is very mature. However, the cost of traditional substrates limits their use in the large-scale development of polylactic acid (PLA) as a green alternative to petroleum-based plastics [3]. Lignocellulosic biomass mainly consisting of cellulose, hemicellulose and lignin is the most abundant renewable carbon source on earth and is inexpensive [4]. It is of great significance to realize the effective biotransformation from lignocellulose-derived sugars to lactic acid. However, owing to its recalcitrance and irregular bond structure, few microorganisms can directly utilize lignocellulose to a product of interest [5]. Thus, pretreatment is indispensable to improve lignocellulose digestibility and release more fermentable sugars for microorganism fermentation [6]. Nevertheless, many pretreatment studies have shown that various inhibitors that severely impede microbial growth and fermentation are inevitably formed during the deconstruction of lignocellulose, mainly organic acids, furan derivatives and phenolic compounds [7]. These inhibitors constitute a severe obstacle to the efficient use of lignocellulosic sugars and the effective production of biochemicals. Therefore, developing low-cost approaches and microorganisms to overcome barriers of inhibitors from biomass for fermentation would contribute to the scale-up and commercialization of lignocellulosic biorefineries.

Currently, a single natural microbial that can detoxify or tolerate the non-sugar ingredients of pretreated lignocellulose hydrolysate while validly transforming sugar mixtures into valuable products is less well reported [8]. Adaptive evolution is considered a powerful tool to improve substrate utilization and tolerance to toxic degradation products of biochemical-producing bacteria [9]. A series of investigations have been conducted to achieve the production of biochemicals from pretreated lignocellulose by using evolved strains [10, 11]. However, the evolution process is time-consuming and labor intensive, and the phenotypes of evolved strains are unstable. In recent decades, genetically engineered hosts that can both tolerate inhibitors and produce a range of different biofuels or biochemicals from lignocellulosic hydrolysate have been reported [12]. The common strategy is the introduction of inhibitor-tolerant elements into product-generating microbes [13, 14]. However, owing to the complexity of hydrolysates and the inherent challenges of engineering and optimizing heterogeneous functional modules in a single strain, low titer products and productivity were obtained by using monocultures based on genetically engineered microorganisms [15]. Alternatively, microbial coculture provides a more feasible and efficacious way to realize this objective [16].

In recent years, synthetic microbial consortia have garnered considerable interest, especially in the field of industrial biotechnology because of their ability to perform complex biotransformation by division of labor [17]. Furthermore, microbial consortia synergies exist that can result in more efficient substrate utilization and increased product yield through functional modularity [18]. The design and establishment of microbial consortia possessing the capability of performing detoxification and biochemical production from pretreated lignocellulosic feedstock hold many appealing properties in practical lignocellulose biorefining [8]. Zhu et al. developed a consortium composed of two *Saccharomyces cerevisiae* strains with specific functions for bioethanol production from pretreated biomass. After process optimization, the ethanol yield of coculture was greatly improved compared with pure culture [19]. Biodetoxification fungus *Amorphotheca resinae* ZN1 coupled with other microorganisms to produce some industrial-related chemicals from dry dilute acid-pretreated corn stover has been reported [20, 21]. In addition, a coculture system of *Acinetobacter baylyi* ADP1 and *Clostridium tyrobutyricum* for the removal of model inhibitors from mock hydrolysate and the production of hydrogen was successfully constructed [22]. These studies have illustrated the potential of coculture strategies in the transformation of lignocellulosic biomass. Despite this progress, they generally demonstrated success with less toxic hydrolysates or displayed potential based on a particular pretreatment method. The development and application of microbial consortia in the effective conversion of pretreated biomass with the high content of inhibitors have thus far been unachieved. In addition, as a prospective approach, reports of microbial consortia with biodetoxification of lignocellulosic hydrolysates and the production of fuels and chemicals are scarce due to the lack of robust biological detoxifying strains.

As a Gram-negative, nonpathogenic soil bacterium, *P. putida* KT2440 has been implicated as a promising new platform organism for biotechnology and synthetic biology applications due to its metabolic and physiologic versatility and flexibility of genetic manipulation [23–25]. Furthermore, this bacterium possesses high resistance to harmful biotechnological related chemicals and to by-products of biomass degradation at concentrations that are inhibitory to other microbial platforms [26, 27]. It exhibits specialized metabolism toward aromatic compounds [28]. These remarkable features of *P. putida* emphasize its potential for the detoxification of lignocellulosic hydrolysates.

**Fig. 1:**
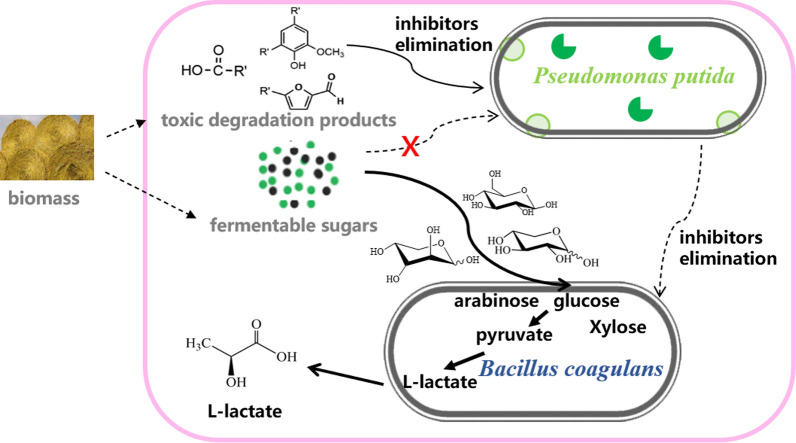
Construction diagram of the microbial coculture system for lactic acid production from lignocellulosic hydrolysate. (Toxic degradation products are divided into three major groups: furan derivatives, organic acids and phenolic compounds; The green dots represent hexoses; The black dots indicate pentoses.)

In this study, a novel synthetic microbial consortium composed of engineered *P. putida* KT2440 and *B. coagulans* NL01 was developed for efficient production of lactic acid from lignocellulosic hydrolysate with a high content of inhibitors (Fig. 1**)**. The sugar catabolism pathway of *P. putida* KT2440 was disabled by deleting the gene encoding glucose dehydrogenase *gcd* and the gene encoding the glucose transporter *gtsABCD* operon from its genome. The engineered strain was expected to concomitantly digest multiple fermentation inhibitors of lignocellulosic hydrolysate while preserving the sugars for lactic acid fermentation by *B. coagulans*. The main purpose of the current work was to provide a strategy of labor division to alleviate the inhibition of fermenting strains caused by mixed inhibitors present in lignocellulosic hydrolysates and to achieve an economically viable process for L-lactic acid production from renewable biomass.

## Results and discussion

### Undetoxified corn stover hydrolysate fermentation by *B. coagulans*

*B. coagulans* as a potential industrial lactic acid-producing strain has received widespread attention. It can effectively convert various monosaccharides (pentose and hexose) derived from lignocellulose into high optical purity L-lactic acid through homolactic fermentation under thermophilic conditions [29]. To exploit the effective utilization of lignocellulosic feedstocks by *B. coagulans*, undetoxified dilute acid-pretreated corn stover hydrolysate consisting of glucose (22.2 g/L), xylose (122 g/L), arabinose (~ 16 g/L), five carbohydrate degradation products, ten phenolic compounds and other unidentified inhibitors (Table 1) was used in this work. The hydrolysate was diluted to different concentrations (20, 30 and 40% (v/v)) and then directly employed for lactic acid fermentation by *B. coagulans* NL01.

**Table 1:** Composition of sugars, weak acids, furans, and phenolic compounds present in concentrated undetoxified dilute-acid pretreated hydrolysate of corn stover

	Products	Concentration
Sugars (g/L)	Glucose	22.36 ± 0.24
	Xylose	121 ± 1.08
	Arabinose	15.20 ± 0.28
Weak acids (g/L)	Formic acid	1.35 ± 0.24
	Acetic acid	14.50 ± 0.25
	Levulinic acid	2.96 ± 0.05
Furans (g/L)	Furfural	0.4 ± 0.16
	HMF	0.99 ± 0.04
Phenolic compounds (mg/L)	3′4-Dihydroxybenzoic acid	34.25 ± 6.02
	4-hydroxybenzoic acid	41.34 ± 0.99
	Vanillic acid	66.35 ± 5.33
	Syringic acid	24.36 ± 1.60
	4-hydroxybenzaldehyde	59.35 ± 1.01
	Vanillin	86.65 ± 2.03
	P-coumaric acid	133.90 ± 2.64
	Syringaldehyde	55.29 ± 2.21
	Ferulic acid	214.20 ± 0.99
	Cinnamic acid	0
Total phenolics (g/L)	Total phenolics	5.33

**Fig. 2:**
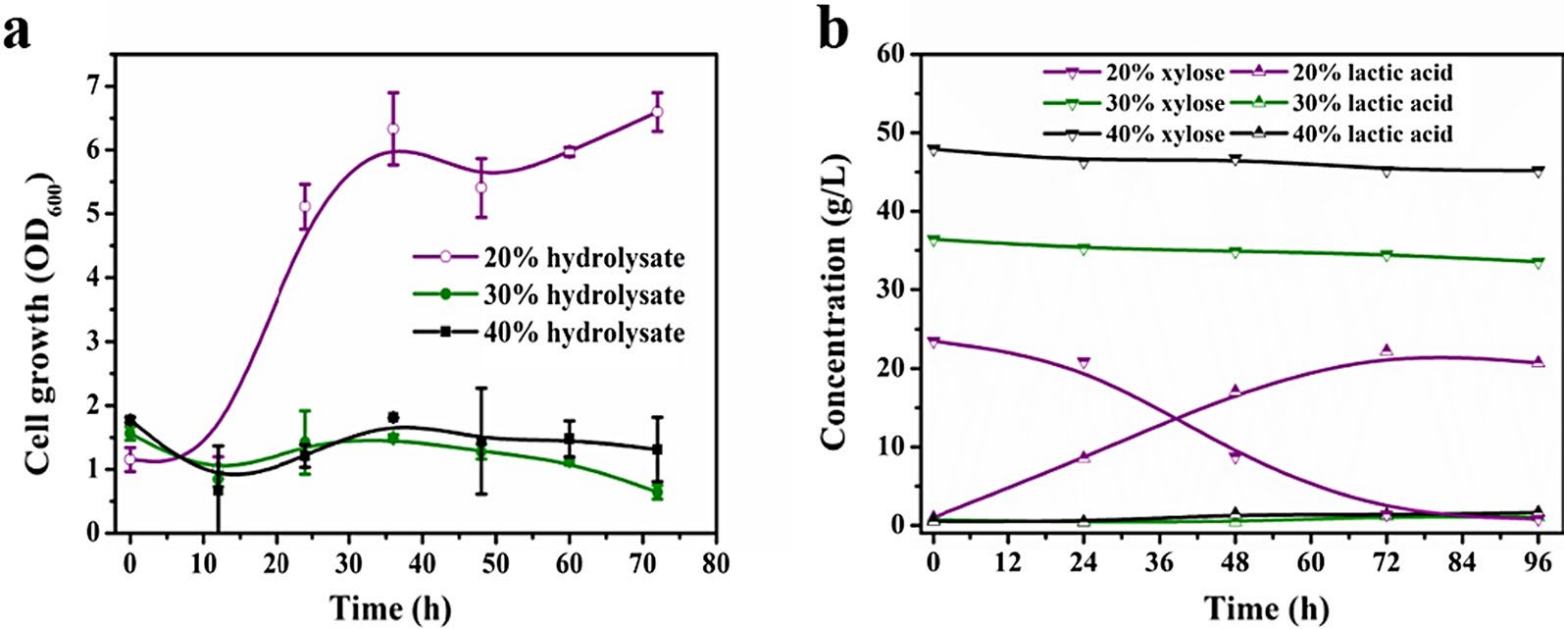
Undetoxified corn stover hydrolysate fermentation by *B. coagulans* NL01. Growth (**a**) and lactic acid fermentation (**b**) of *B. coagulans* NL01 with different initial concentrations of corn stover hydrolysate. Data shown as mean ± SD from at least two independent experiments

The growth and lactic acid fermentation capacity of *B. coagulans* in fermentation medium containing different concentrations of hydrolysate were evaluated. As illustrated in Fig. 2a and b, the growth and xylose utilization of *B. coagulans* decreased as the concentration of the hydrolysate was elevated. At a concentration of 20% (v/v) hydrolysate with 31.93 g/L monomeric sugars, the bacteria showed obvious growth after an initial 12 h lag phase. Primary monosaccharides composed of xylose, glucose and arabinose were completely taken up within 72 h and achieved a maximum OD_600_ of 6.6. However, the growth and lactic acid fermentation of *B. coagulans* were completely ceased at a higher hydrolysate concentration (30% (v/v)). Even after prolonged fermentation time to 96 h, the growth of *B. coagulans* was still seriously hindered. The same results were observed with 40% (v/v) hydrolysate.

These results demonstrated that *B. coagulans* had a certain tolerance to hydrolysate, but the tolerance ability of the strain was not high. It was merely suited for hydrolysates with low inhibitors and low corresponding sugar concentrations. It is well known that substrate titer is a of vital factor impacting the final product concentration [30]. However, the concentration of inhibitors in the hydrolysate increased with increasing sugar concentration. Furthermore, synergistic effects between inhibitors at high concentrations render lignocellulosic hydrolysates more toxic. The poor fermentation performance indicated that the tolerance of *B. coagulans* to higher concentration of hydrolysate was very inferior. These features limit the potential of using this bacterium as a platform strain to process the carbohydrate products of lignocellulosic waste [31]. To promote the effective conversion of lignocellulosic sugars in hydrolysates, the introduction of an outstanding detoxified strain capable of transforming and degrading inhibitors while preserving sugars is highly desirable for removing inhibitory and toxic compounds to improve the fermentation of *B. coagulans.*

### Metabolic engineering of *P. putida* to block sugar metabolism and growth characterization of knockout strains

**Fig. 3:**
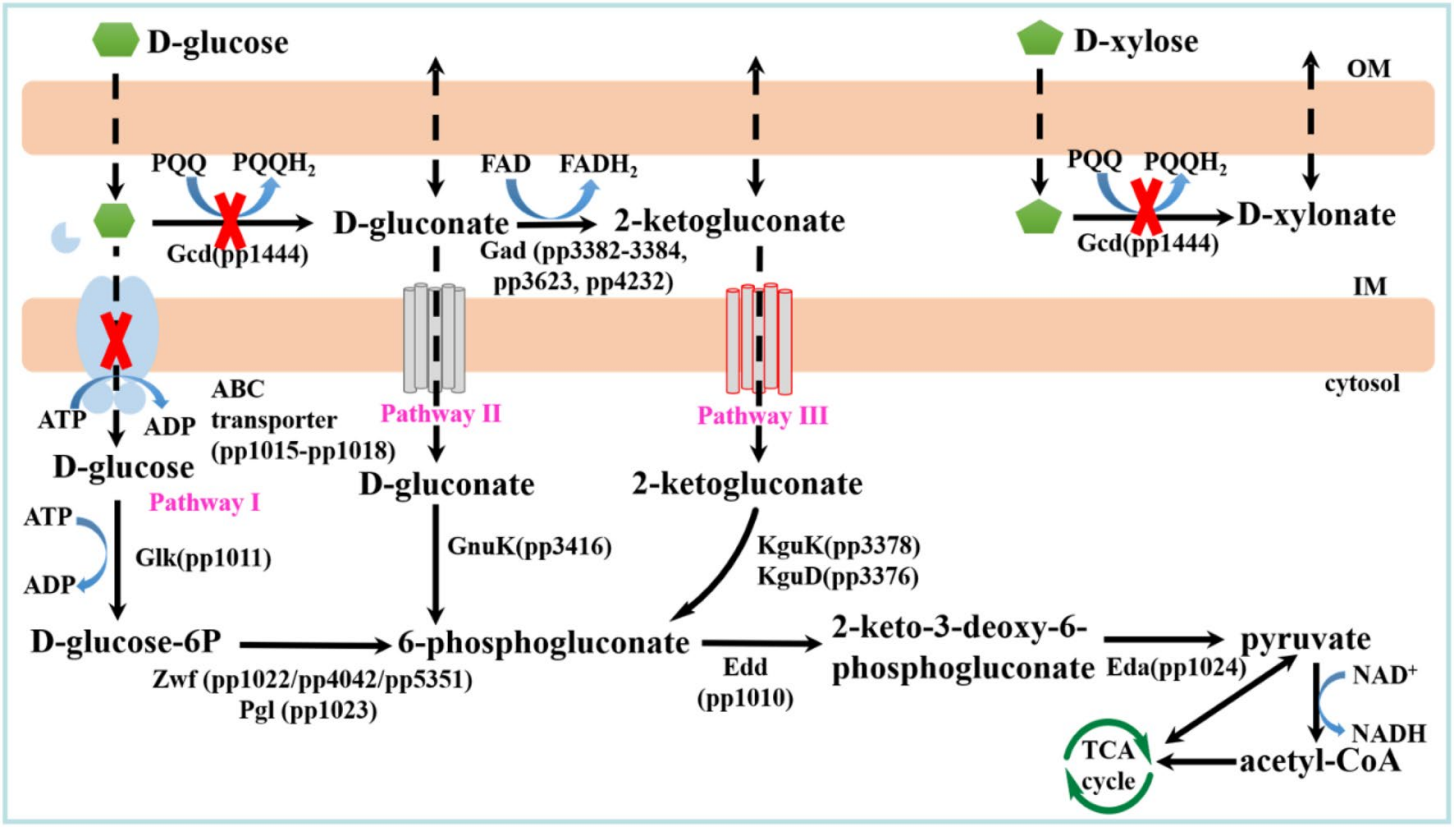
Schematic representation of glucose metabolism and xylose transformation in *P. putida* KT2440. Glucose catabolism in *P. putida* occurs through the simultaneous operation of three pathways that converge at the level of 6-phosphogluconate. D-xylose was oxidized to the dead-end product D-xylonate by the periplasmic glucose dehydrogenase Gcd instead of being used as a carbon source for growth. Abbreviations used for the metabolic intermediates and enzymes within the biochemical network are as follows: *PQQ* pyrroloquinoline quinone; *Gcd* glucose dehydrogenase, *FAD* flavin adenine dinucleotide, *Gad* gluconate dehydrogenase, *Glk* glucokinase, *GnuK* gluconate kinase,* KguK* 2-ketogluconate kinase, *KguD* 2-ketogluconate-6-phosphate reductase, *Zwf* glucose-6-phosphate dehydrogenase, *Pgl* 6-phosphogluconolactonase, *Edd* 6-phosphogluconate dehydratase, *Eda* 2-keto-3-deoxy-6-phosphogluconate aldolase, *TCA cycle* tricarboxylic acid cycle, *OM* outer membrane, *IM* inner membrane

*P. putida* KT2440 has emerged as a promising candidate for a future lignocellulose‐based biotechnology process due to its outstanding toxicity tolerance of numerous byproducts of biomass hydrolysis [32, 33]. This was confirmed here by incubating wild-type *P. putida* in LB medium supplemented with major inhibitors found in lignocellulosic hydrolysate (Additional file 1: Fig. S1). In addition, it is a robust microorganism capable of natively catabolizing glucose other than toxins. Unfortunately, the excessive utilization of sugar during detoxification will adversely affect subsequent fermentation and reduce process efficiency. With the intent of developing a prominent detoxified strain capable of removing inhibitors in hydrolysate and retaining monomeric sugars, we engineered *P. putida* to delete its sugar metabolism. There are three glucose catabolism pathways in *P. putida* KT2440 (Fig. 3). In the first route, glucose is transported to the cytoplasm from the periplasm by means of an ATP-dependent ABC transporter, encoded by the *gtsABCD* operon (PP_1015-PP_1018), and is then phosphorylated by glucokinase to glucose-6-phosphate and subsequently converted by glucose-6-phosphate dehydrogenase and 6-phosphogluconolactonase to 6-phosphogluconate. The second is a periplasmic oxidation pathway mediated by PQQ-dependent glucose dehydrogenase *gcd* (PP_1444). Glucose is oxidized to gluconate by *gcd* in the periplasm. Gluconate is then transported to the cytoplasm and subsequently phosphorylated by gluconokinase to 6-phosphogluconate. In addition, previous studies revealed that xylose is also oxidized to xylonate by *gcd* [26]. The third route is the 2-ketogluconate loop, which involves the oxidation of gluconate to 2-ketogluconate. The resulting 2-ketogluconate is imported into the cytoplasm via the outer membrane and subsequently phosphorylated by 2-ketogluconate kinase into 2-keto-6-phosphogluconate, and later reduced to 6-phosphogluconate via 2-ketogluconate-6-phosphate reductase. The oxidation pathway and the direct phosphorylation route converge at the node of 6-phosphogluconate and that function simultaneously [26, 34]. Accordingly, *gcd* and *gtsABCD* were selectively removed.

**Fig. 4:**
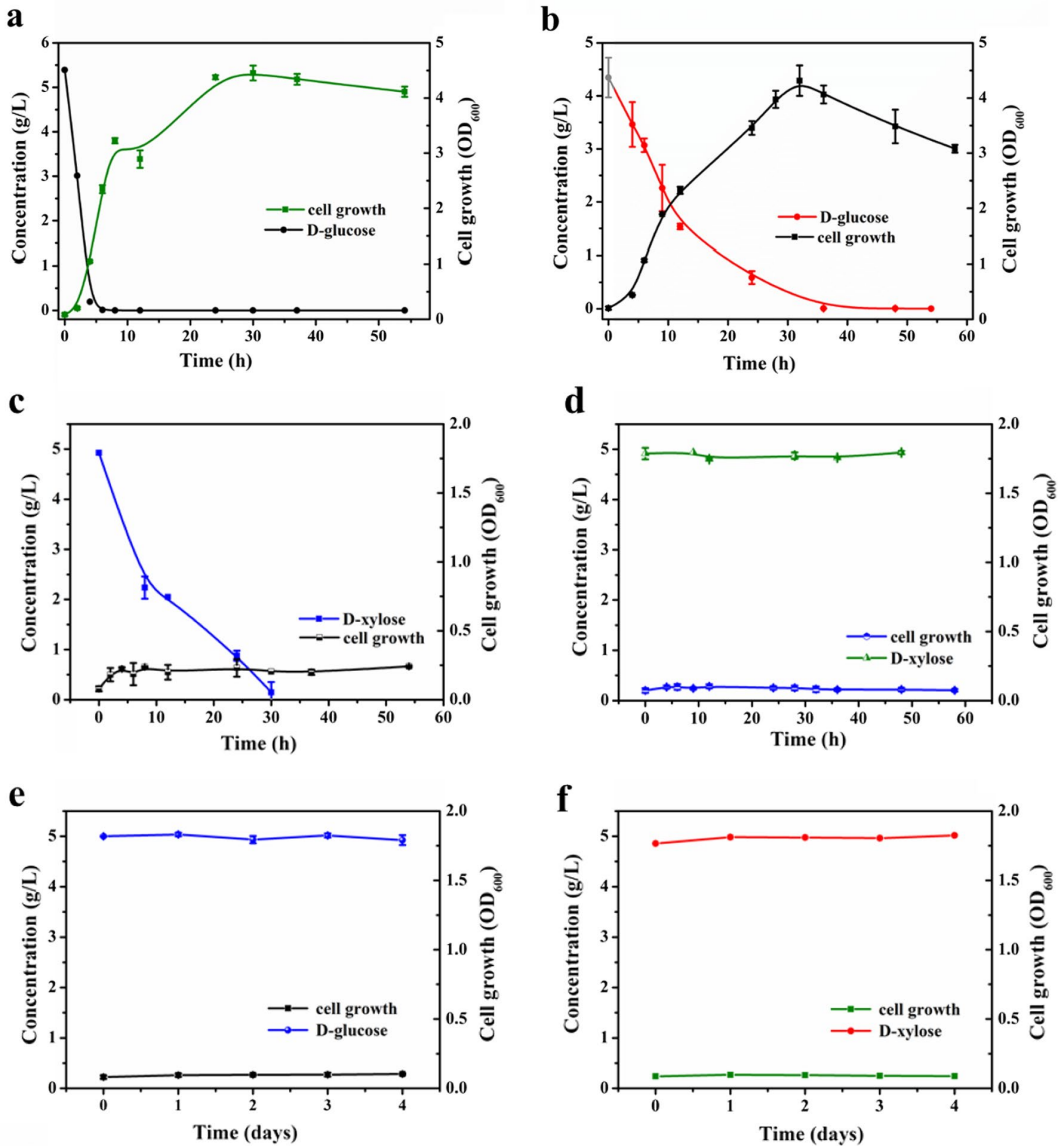
Growth characterization of wild-type *P. putida* KT2440 and its knockout strains in M9 minimal medium. *P. putida* KT2440 (**a**) and *P. putida* KT2440 pK18MS-Δ*gcd* (**b**) with 5 g/L D-glucose; *P. putida* KT2440 (**c**) and *P. putida* KT2440 pK18MS-Δ*gcd* (d) with 5 g/L D-xylose; *P. putida* KT2440 pK18MS-Δ*gcd*-Δ*gtsABCD* with 5 g/L D-glucose (**e**) and 5 g/L D-xylose (**f**). Data shown as mean ± SD from at least two independent experiments

The growth and consumption of fermentable sugars of *P. putida* and its knockout strains were characterized. As a reference, cultivation of *P. putida* KT2440 in M9 minimal medium using glucose or xylose as a carbon source was performed. As shown in Fig. 4a, approximately 5 g/L glucose could be used up by *P. putida* KT2440 in 6 h and reached a remarkable maximum cell OD_600_ of 4.5. However, when the carbon source of *P. putida* KT2440 was switched to xylose, although the xylose concentration was significantly reduced and dropped to 0 at 30 h, the growth of the strain was not observed (Fig. 4c). The consumed xylose was oxidized to xylonate instead of being used as a carbon source for strain growth. The results obtained are consistent with a previous study [35]. The growth performance of *P. putida* pK18MS-Δ*gcd* was evaluated under the same carbon source conditions. When incubated with glucose, *P. putida* KT2440 pK18MS-Δ*gcd* showed significantly slower growth than the wild type and the consumption rate of glucose was remarkably decreased, and was not fully consumed until 36 h (Fig. 4b). The ability of *P. putida* KT2440 pK18MS-Δ*gcd* to utilize xylose was also tested (Fig. 4d). No growth of the strain or reduced sugar concentration could be examined during the entire cultivation. The deletion of *gcd* could greatly retard glucose metabolism and completely hinder the consumption of xylose. These results suggested that *gcd* is of paramount importance for glucose and xylose uptake in *P. putida*.

The successful knockout strain *P. putida* KT2440 pK18MS-Δ*gcd*-Δ*gtsABCD* was verified using glucose or xylose as a substrate. As predicted, the strain did not show any detectable growth or consumption of sugar during 4 days of cultivation on glucose or xylose (Fig. 4e & f). Removal of *gcd* and *gtsABCD* from the *P. putida* KT2440 genome rendered it unable to utilize glucose and xylose.

### Inhibitor tolerance analysis of engineered *P. putida* KT2440 pK18MS-Δ*gcd*-Δ*gtsABCD*

**Fig. 5:**
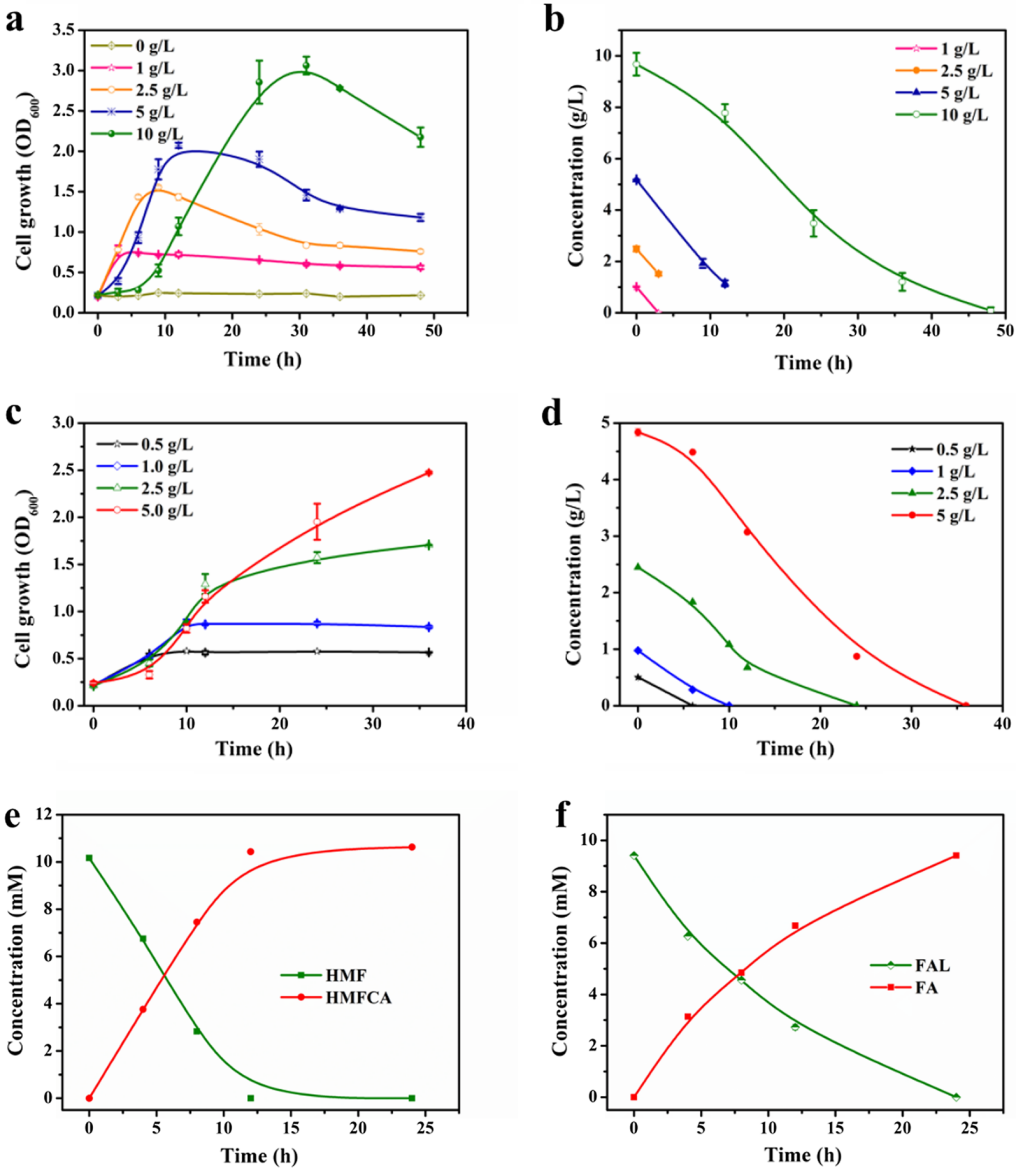
Organic acids and furan aldehydes tolerance analysis of engineered *P. putida* KT2440 pK18MS-Δ*gcd*-Δ*gtsABCD* in M9 minimal medium. The cell growth (**a**) and depletion (**b**) with different concentrations of sodium acetate; The cell growth (**c**) and depletion (**d**) with different concentrations of levulinic acid; The conversion of HMF (**e**) and FAL (**f**). Data shown as mean ± SD from at least two independent experiments

The ability of engineered *P. putida* KT2440 pK18MS-Δ*gcd*-Δ*gtsABCD* to metabolize and convert common inhibitors encountered in lignocellulosic hydrolysates was investigated. First, the effect of acetate, which was formed as a major unwanted byproduct from the breakdown of hemicellulose, was tested by culturing cells in M9 minimal medium comprising different titers of sodium acetate ranging from 0–10 g/L. As presented in Fig. 5a, the knockout strain could still grow well with different concentrations of sodium acetate as a carbon source. Supplementation with high concentrations of sodium acetate (5 and 10 g/L) only slightly delayed the growth of cells in the early stage but did not affect their complete catabolism by the strain. In addition, the maximum achieved biomass of the strain was positively correlated with the concentration of the initial substrate provided. With 1 g/L sodium acetate, the maximum OD_600_ of 0.77 was detected. Using 5 g/L sodium acetate, a higher growth rate was measured. The cells reached a maximum OD_600_ of 3.1 at the highest applied concentration of 10 g/L sodium acetate. During cultivation, the consumption of sodium acetate at different concentrations is also indicated in Fig. 5b. Up to 10 g/L sodium acetate could be totally depleted by engineered *P. putida* in 48 h. Although acetate itself is not a strong inhibitor, its elimination lessens the overall toxicity of hydrolysate [22]. Similarly, the utilization of levulinic acid by the knockout strain was assessed. The knockout strain did not exhibit lag phase at the tested concentrations (Fig. 5c). The strain could grow rapidly with levulinic acid. A maximum OD_600_ of 2.5 could be achieved starting at 5 g/L levulinic acid. Correspondingly, the consumption of levulinic acid can be seen from Fig. 5d. Levulinic acid was rapidly consumed in a short period. Although previous studies have reported that weak acids can inhibit cell growth and metabolism due to uncoupling and intracellular anion accumulation [36], we found that engineered *P. putida* grew well in the presence of carbohydrate-derived acids. Furfural (FAL) and 5-hydroxymethylfurfural (HMF), which are commonly generated from high-temperature processing of lignocellulose are considered major inhibitors in microbial conversion processes [37]. The conversion performance of the engineered *P. putida* KT2440 pK18MS-Δ*gcd*-Δ*gtsABCD* toward furan aldehydes was examined. As shown in Fig. 5e and f, the engineered strain could convert the representative concentrations of HMF and FAL into the corresponding carboxylic acids, 5‑hydroxymethyl‑2‑furancarboxylic acid (HMFCA) and furoic acid (FA), which are less toxic. In addition, a different conversion profile of engineered *P. putida* was observed. Compared with FAL, the conversion rate of HMF was faster within 12 h (0.847 mM/h for HMF and 0.557 mM/h for FAL). The complete conversion of HMF was observed at 12 h of cultivation, whereas full conversion of FAL was observed at 24 h.

**Fig. 6:**
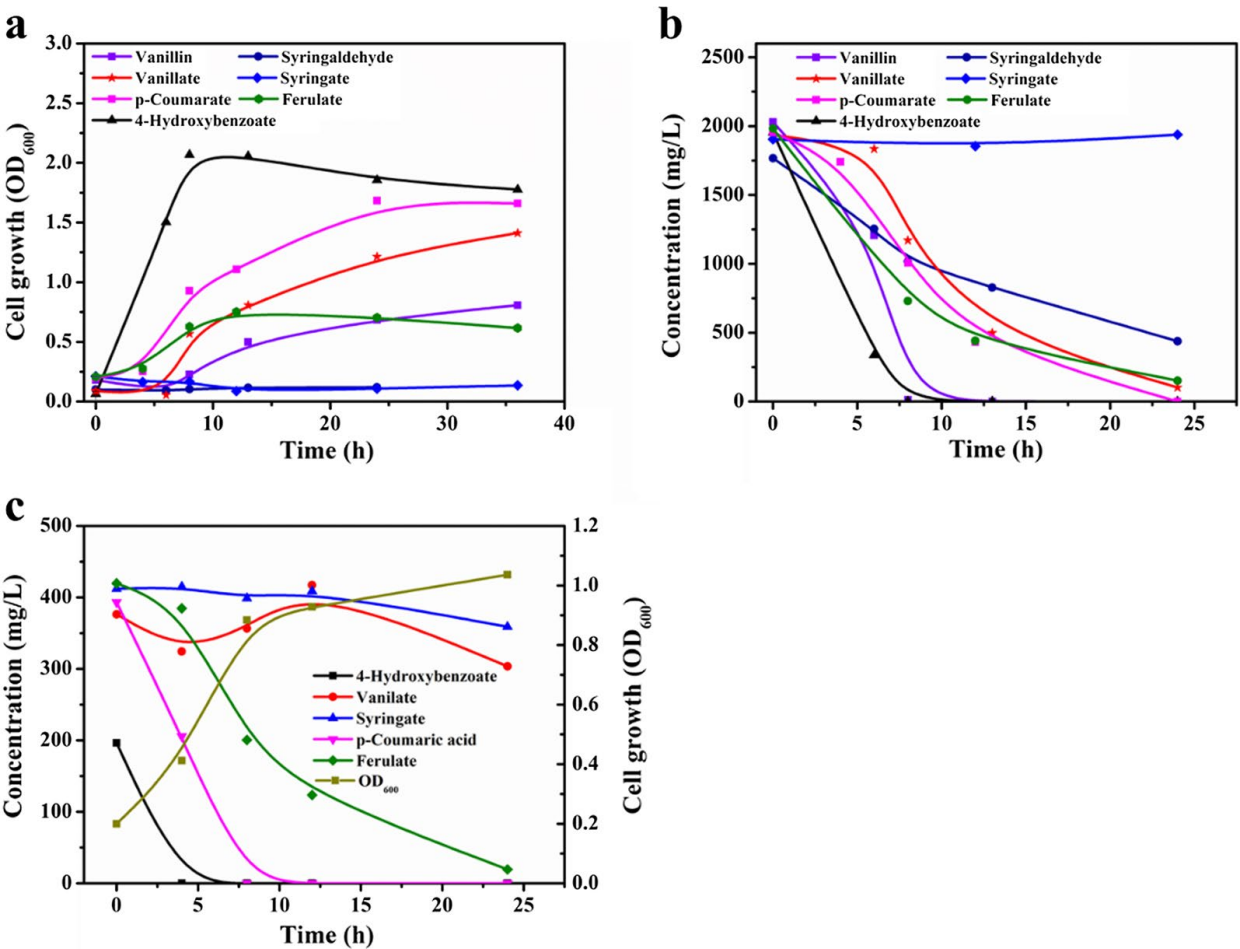
Phenolics analysis of engineered *P. putida* KT2440 pK18MS-Δ*gcd*-Δ*gtsABCD* in M9 minimal medium. The cell growth (**a**) and depletion (**b**) with each approximately 2 g/L of either ferulate, p-coumarate, 4-hydroxybenzoate, syringate, vanillate, vanillin, syringaldehyde; The cell growth and depletion with a mixture of ferulate, p-coumarate, 4-hydroxybenzoate, syringate and vanillate (**c**)

Phenolic compounds generated from lignin breakdown exhibited severe toxic inhibition on the growth and fermentation of most microorganisms [38]. The performance of engineered *P. putida* KT2440 to metabolize aromatic compounds was characterized by incubating the strain in minimal medium with each approximately 2 g/L of phenolic compound. The cell density and substrate concentration were measured (Fig. 6a and b) during cultivation. The engineered strain behaved a robust growth with the major these compounds as the sole carbon source. The strain consumed 1.95 g/L 4-hydroxybenzoate in 8 h, yielding an OD_600_ of 2.1. The equal concentration of p-coumarate was metabolized by engineered *P. putida* in 24 h, achieving an OD_600_ of 1.68. Vanillin was fully converted into vanillate in 8 h and then used for cell growth. No growth of engineered *P. putida* KT2440 was observed when using syringaldehyde or syringate as the sole carbon source, despite a significant reduction of syringaldehyde was detected. Syringate concentrations determined in medium with syringaldehyde as the single carbon source suggested that syringaldehyde was converted to the syringate, which was incapable of being used for biomass formation. When a mixture thereof five phenolic acids compounds are present (Fig. 6c). We observed no delay in bacterial growth. The engineered *P. putida* KT2440 can grow well via consuming these compounds almost simultaneously. The strain exhausted 196.2 mg/L of 4-hydroxybenzoate within 4 h and consumed 393.1 mg/L of p-coumarate within 8 h. 420 mg/L of ferulate nearly was exhausted in 24 h. However, the metabolism of ferulate enabled the modest accumulation of vanillate. The syringate cannot be used as a carbon source for growth but can still be degraded slowly by exponential phase cells.

These results demonstrated that genetically modified *P. putida* remained robust to the elimination and transformation of toxic inhibitors. It has extraordinary potential for the removal of inhibitors in pretreated biomass.

### Production of lactic acid from undetoxified corn stover hydrolysate by using synthetic consortium containing engineered *P. putida* and *B. coagulans*

**Fig. 7:**
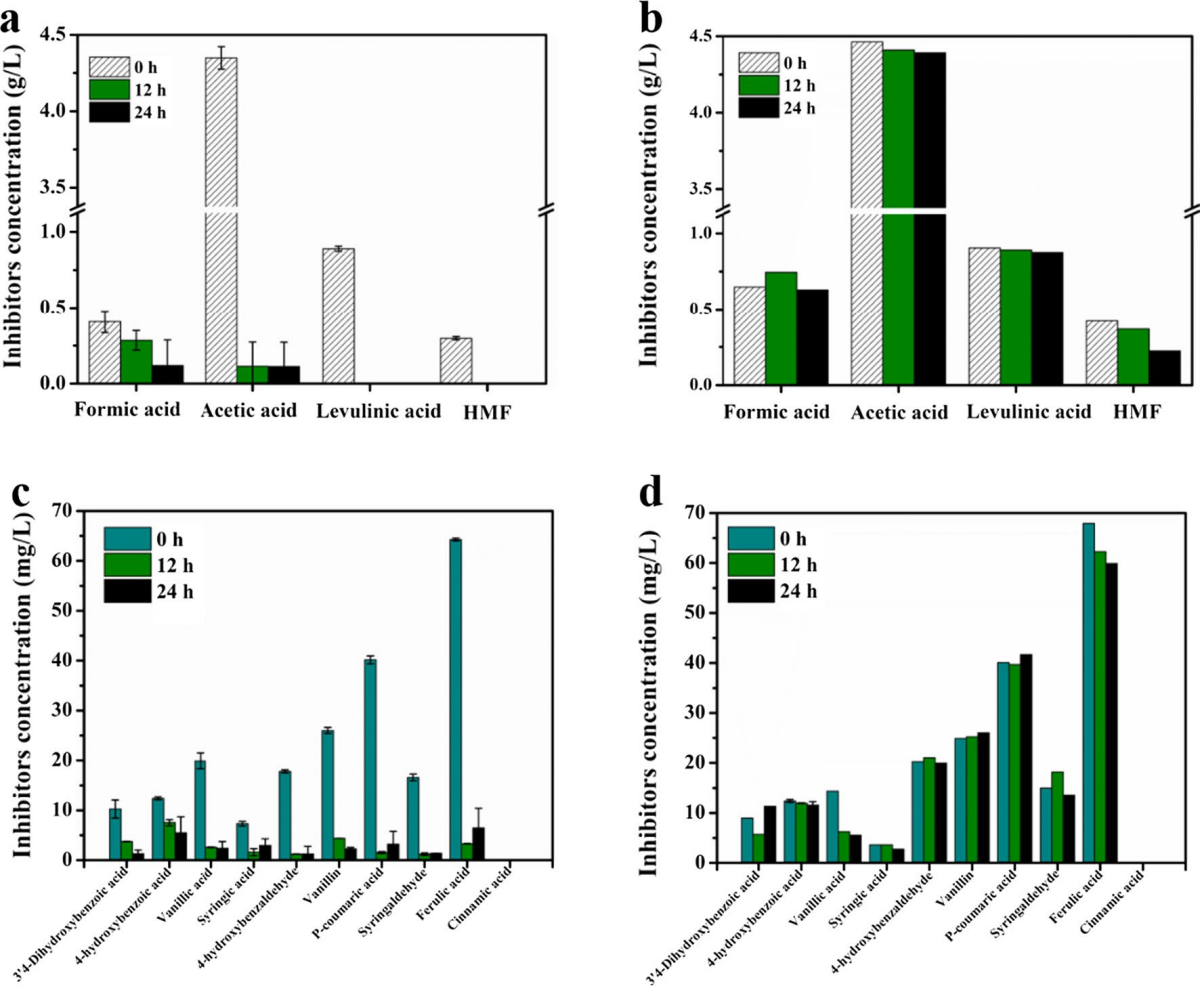
Comparison of the profiles of inhibitors in undetoxified 30% (v/v) hydrolysate with engineered *P. putida* KT2440 pK18MS-Δ*gcd*-Δ*gtsABCD* and without detoxified strain (control). The measured parameters include the main inhibitors. The profiles of organic acids and HMF with engineered *P. putida* (**a**) and without engineered *P. putida* (**b**); The profiles of phenolic compounds with engineered *P. putida* (**c**) and without engineered *P. putida* (**d**)

Based on the above results, a cocultivation reaction was performed for L-lactic acid production from undetoxified 30% (v/v) real hydrolysate that has the same composition as described above. During coculture process, to gain detailed insights into the behavior of engineered *P. putida* KT2440 pK18MS-Δ*gcd*-Δ*gtsABCD* in complex mixtures of the hydrolysate, the detoxification reaction of the hydrolysate was set up using 1 g/L *P. putida* KT2440 pK18MS-Δ*gcd*-Δ*gtsABCD*, and no detoxified strain was supplemented as a control. The concentration changes of substances (fermentable sugars, organic acids, furans and phenol compounds) before and after the reaction were detected. After 24 h of reaction, the concentrations of major sugars such as glucose, xylose and arabinose in lignocellulosic hydrolysate remained nearly constant under both conditions (Additional file 1: Table S1). However, there were considerable differences in the degradation and conversion of inhibitors. In the presence of the detoxified strain, 4.3 g/L acetic acid and 0.9 g/L levulinic acid could be completely metabolized in 12 h. In addition, the removal rates of formic acid and HMF reached 73% and 100%, respectively (Fig. 7a). Furthermore, the concentration changes of total phenol and several typical monophenol inhibitors were also quantified. As shown in Additional file 1: Table S2 and Fig. 7c, we observed that the concentrations of total phenol and monophenol inhibitors greatly reduced. After detoxification for 24 h by *P. putida* KT2440 pK18MS-Δ*gcd*-Δ*gtsABCD*, 25% of the total phenolic derivatives were removed. This result is comparable to the electrochemical detoxification reported in the literature [30]. In addition, most of the representative monophenol compounds, especially ferulic acid, *p*-coumaric acid, vanillin, 4-hydroxybenzaldehyde and syringaldehyde, could be rapidly degraded to very low concentrations. A control experiment confirmed that the concentrations of organic acids, furan aldehydes (Fig. 7b), ten representative phenolic compounds (Fig. 7d) and total phenolic derivatives (Additional file 1: Table S2) did not vary significantly in the absence of detoxified strain pK18MS-Δ*gcd*-Δ*gtsABCD* under the same investigated conditions. These results indicated that *P. putida* KT2440 pK18MS-Δ*gcd*-Δ*gtsABCD* is a superior biological detoxification strain that can effectively eliminate toxic compounds in depolymerized lignocellulose streams while retaining hexose and pentose sugars in the hydrolysate for subsequent lactic acid fermentation.

**Fig. 8:**
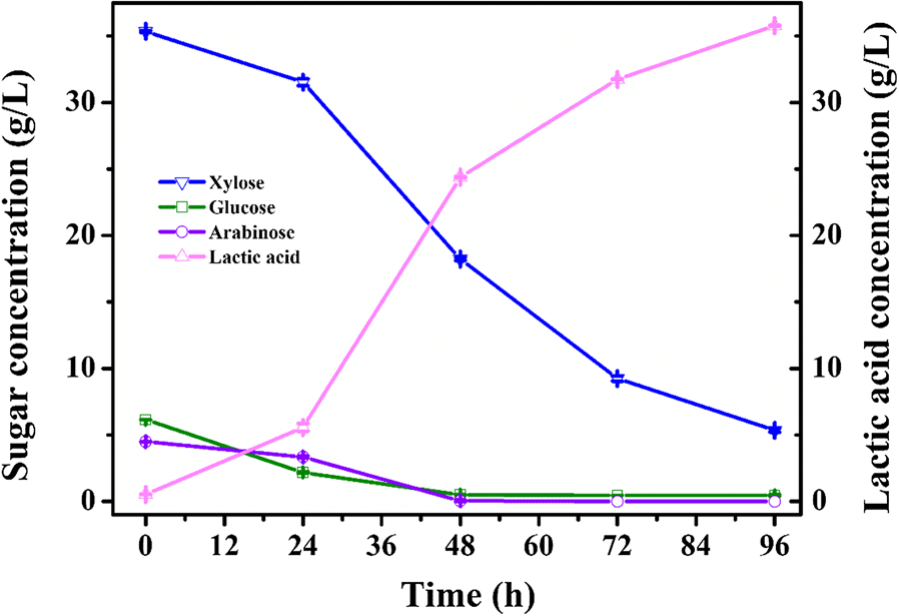
Production of lactic acid from undetoxified 30% (v/v) lignocellulosic hydrolysate by a two-stage sequential scheme

The feasibility of detoxified hydrolysate for lactic acid fermentation by *B. coagulans* was investigated. As shown in Fig. 8, the 30% (v/v) hydrolysate treated by *P. putida* KT2440 pK18MS-Δ*gcd*-Δ*gtsABCD* containing glucose (6.1 g/L), xylose (approximately 35 g/L) and arabinose (4.5 g/L) could be effectively consumed by fermentation of strain *B. coagulans* within 96 h, resulting in lactic acid production of 35.8 g/L and a corresponding yield of 0.8 g/g. However, hydrolysate at the identical concentration that were not detoxified by *P. putida* KT2440 pK18MS-Δ*gcd*-Δ*gtsABCD* were unable to grow and were effectively fermented by *B. coagulans* (Fig. 2). As anticipated, the degradation and transformation of diverse inhibitors by *P. putida* in hydrolysate greatly decreased the inhibitor concentration and contributed to the broth being less toxic for fermentation. The results suggested that the detoxification of *P. putida* was extremely beneficial for improving the utilization of sugar and lactic acid fermentation by *B. coagulans* in hydrolysates with a high content of complex inhibitors. In addition, the coculture system was more advantageous than monoculture fermentation for the production of biochemicals from highly toxic lignocellulosic hydrolysate.

### Comparison of lactic acid production from lignocellulosic biomass with other studies

To date, extensive investigations have been conducted to realize the production of lactic acid from lignocellulosic biomass (Table 2). The production of lactic acid from pretreated wheat straw by adapted *B. coagulans* was reported. Lactic acid (35.5 g) was obtained from 80 g of wheat straw with a yield of 0.44 g/g [39]. In addition, to reduce processing costs and simplify of the process, one genetically engineered *Bacillus subtilis* capable of both degrading cellulose and producing lactic acid was developed. Nevertheless, recombinant cellulolytic *B. subtilis* suffered from low titers of lactic acid [40]. Low efficiency was the main barrier for lactic acid production from lignocellulosic biomass. Most studies on lactic acid production from lignocellulosic biomass use the simultaneous saccharification and fermentation (SSF) method. Cui et al. reported mixed cultures of *Lactobacillus rhamnosus* and *Lactobacillus brevis* for bioconversion of corn stover into lactic acid through the SSF process [41]. Although a relatively high productivity of lactic acid was obtained, it requires the addition of high-cost cellulase. Recently, consolidated bioprocessing (CBP) targeting lactic acid production was exploited by using a synthetic microbial consortium of *Trichoderma reesei* and *Lactobacillus pentosus*. Ultimately, 34.7 g/L and 19.8 g/L lactic acid were obtained from 5% (w/w) microcrystalline cellulose and pretreated beech wood, respectively [42]. Despite intensive research efforts spanning years, as an immature but promising technology, there are limited reports on the conversion of lignocellulose by microbial cocultivation especially in lignocellulosic hydrolysates made up of diverse inhibitors and high concentrations. In this work, we developed synthetic microbial consortium of *P. putida* and *B. coagulans* that could achieve effective production of lactic acid from lignocellulosic hydrolysate with high inhibitor contents. The titer and yield of lactic acid attained by the coculture system were comparable to those previously reported. Although the results obtained by this approach are encouraging, the process regulation and optimization of microbial cocultivation are still essential in future work, which will promote the concentration and productivity of lactic acid to satisfy the requirements of practical lignocellulose biorefinement.

**Table 2:** Comparison of lactic acid production from lignocellulosic biomass with other studies

Strain	Strategy	Substrate	CLA, max (g L^−1^)	Yield (g/g)	Productivity (g L^−1^ h^−1^)	References
*B. coagulans* CC17A	Adaptive evolution, SSF	Wheat straw	35.5	0.44 ^a^	n.a.^c^	[39]
*B. subtilis*	Over-expression of endoglucanase and knocked out alpha-acetolactate synthase	Cellulose	4.1	0.58^a^	0.03	[40]
*L. rhamnosus*/*L. brevis*	Coculture, SSF	Corn stover	20.95	0.70^a^	0.58	[41]
*T. reesei*/*L. pentosus*	CBP	Microcrystalline cellulose	34.7	0.62^b^	0.16	[42]
*T. reesei*/*L. pentosus*	CBP	Beech wood	19.8	0.78^b^	0.10	[42]
*P. putida/B. coagulans*	Sequential coculture	Corn stover hydrolysate	35.8	0.80^b^	0.37	This study

^a^Lactic acid yield was based on the total weight of substrate added

^b^Lactic acid yield was based on total fermentable sugars

^c^n.a.: not available

## Conclusions

In this study, a synthetic consortium was constructed for converting highly toxic lignocellulosic hydrolysate into lactic acid. The consortium consisted of engineered *P. putida* capable of consuming and transforming inhibitors while leaving the lignocellulosic sugars intact for subsequent fermentation and *B. coagulans* capable of utilizing fermentable sugars to produce lactic acid*.* Compared with the fermentation of undetoxified hydrolysate by monoculture without lactic acid production, a microbial coculture generated 35.8 g/L lactic acid with a yield of 0.8 g/g total sugars from an equal titer of hydrolysate. The results herein affirmed the potential of using synthetic consortia to compartmentalize biological functions into specific strains to accomplish complicated tasks that are difficult to achieve with monocultures.

## Methods

### Materials

Corn steep liquor was kindly donated by Shandong Xinpu Biotechnology Co., Ltd (Shandong, China). Concentrated undetoxified dilute-acid pretreatment corn stover hydrolysate was kindly provided by Shanghai Yigao Biotechnology Co., Ltd (Shanghai, China). *P. putida* KT2440 and *B. coagulans* NL01 were obtained from the Biochemical Engineering Research Institute of Nanjing Forestry University. FAL (99%) and HMF (99%) were purchased from Sigma–Aldrich. Levulinic acid (> 97%) was purchased from TCI (Japan).

### Plasmid and strain constructions

The construction of *gcd* and *gtsABCD* mutants was conducted as described in our previous study [43]. Specifically, genomic DNA of *P. putida* KT2440 was isolated using a Bacteria Genomic DNA Extraction Kit (Takara, Japan). The upstream and downstream homologous arms of the *gcd* and *gtsABCD* genes were separately PCR-amplified using genomic DNA from *P. putida* KT2440 as the template and their respective primers (Additional file 1: Table S3). After gel purification, overlap extension PCR of *gcd* and *gtsABCD* homologous arms was executed using the primers *gcd*-up.f and *gcd*-down.r or *gtsABCD*-up.f and *gtsABCD*-down.r. The resulting PCR products were recovered by a gel and PCR clean-up system (Promega, USA) and ligated into the pEASY-Blunt cloning vector to form new plasmids. The plasmids were digested with *Eco*R I and *Bam*H I and subsequently cloned into the corresponding restriction sites of the suicide vector pK18mobsacB to yield the recombinant plasmids pK18MS-Δ*gcd* and pK18MS-Δ*gtsABCD*. This was followed by electroporation of the plasmid pK18MS-Δ*gcd* into electrocompetent *P. putida* KT2440. Single crossover and double-crossover transformants of *P. putida* KT2440 pK18MS-Δ*gcd* were selected separately on LB plates with 50 μg/mL kanamycin and LB agar plates supplemented with 15% (w/v) sucrose and incubated at 30 °C. The correct double-crossover recombinant of *P. putida* KT2440 pK18MS-Δ*gcd* was used as the starting strain for further construction of *gtsABCD* knockout bacteria. The knockout process was the same as that described above.

### The growth and transformation of sugars, organic acids, furan aldehydes and phenols by *P. putida*

*P. putida* KT2440 and its knockout strains were routinely precultured overnight in 5 mL of Luria broth (LB) medium at 30 °C and 200 rpm. Then, the overnight cultures were collected by centrifugation at 6000 g for 5 min, and the supernatants were removed. The recovered cells were washed twice with 0.85(w/v) NaCl. Then, the cells were inoculated at an OD_600_ of 0.1–0.2 in a 250‐mL shake flask with 25 mL of M9 minimal medium [26] containing different substrates. The concentrations of added glucose and xylose are depicted in the text or corresponding figure caption. In addition, acetate, levulinic acid, FAL, HMF, ferulic acid, p-coumaric acid, 4-hydroxybenzoic acid, syringate, vanillic acid, vanillin, and syringaldehyde were added to the medium at specified concentrations. After the addition of the inhibitors, the initial pH of the medium was adjusted to 7.0 using NaOH solution.

### Biodetoxification of lignocellulosic hydrolysate by engineered *P. putida*

The engineered *P. putida* KT2440 pK18MS-Δ*gcd*-Δ*gtsABCD* was preactivated overnight. Then, 2% seed cultures were inoculated into 200 mL of LB medium in a 1 L shake flask and incubated for approximately 12 h. The cells were centrifuged (6000 g, 5 min), and pellets were washed with 0.85(w/v) NaCl solution twice. Subsequent harvested cells were used for biodetoxification. Biodetoxification was performed in an Erlenmeyer flask with 50 mL of hydrolysate-containing fermentation medium (pH 7.0) without a nitrogen source supply and 1 g/L inoculum consisting of *P. putida* KT2440 pK18MS-Δ*gcd*-Δ*gtsABCD* cells at 30 °C and 200 rpm under aerobic conditions.

### Fermentation experiments with the *B. coagulans* strain

*B. coagulans* NL01 was used for lactic acid fermentation. Typically, the preculture of *B. coagulans* NL01 was conducted at 50 °C and 150 rpm for 12 h in seed medium containing (g/L) xylose 20, yeast extract 1, corn steep powder 2.5, NH_4_Cl 1, MgSO_4_ 0.2, and CaCO_3_ 10 and then transferred into the corresponding hydrolysate-containing fermentation medium with a 10% (v/v) inoculum for lactic acid production. Fermentation experiments were performed in a 150-mL shake-flask containing 50 mL of fermentation medium. The samples were withdrawn at regular intervals to determine the amounts of residual sugars and lactic acid produced during the reaction. The hydrolysate-containing medium used for *B. coagulans* fermentation was prepared as follows: concentrated undetoxified dilute-acid pretreated hydrolysate of corn stover was first neutralized to pH 6.5. Then, the hydrolysate was centrifuged (10,000 g, 15 min), and the supernatant was added to the fermentation medium [39] of *B. coagulans* deprived of carbon source, and the initial pH was controlled to 7.2.

### The process for the production of lactic acid from lignocellulosic hydrolysate by a two-stage sequential scheme

A two-stage biological process for the detoxification of hydrolysate and the generation of lactic acid was performed as follows. Detoxification using *P. putida* KT2440 pK18MS-Δ*gcd*-Δ*gtsABCD* to degrade and convert inhibitors of hydrolysate was conducted at 30 °C at 200 rpm for 24 h under aerobic conditions. After that, the pH of hydrolysate was monitored and adjusted to 7.2 with 5 M HCl. Following *B. coagulans* cells at 10% of the inoculum, organic nutrients (yeast extract (2.5 g/L) and corn steep power (1.2 g/L)) as well as CaCO_3_ (approximately half of the total sugar concentration) were added to the hydrolysate for lactic acid fermentation. The reaction was shaken in an incubator at 50 °C and 150 rpm.

### Analytic methods

All concentrations of the monomeric sugars, organic acids, furan aldehydes and lactic acid were detected by an Agilent 1260 HPLC system with a reflective index detector. The analysis was conducted using an Aminex HPX-87H column (Bio–Rad, USA) operated at 55 °C, and the eluent flow rate was 0.6 mL/min with 5 mM H_2_SO_4_, unless otherwise stated. The concentrations of phenolic inhibitors were determined as previously reported by Ouyang et al. [39]. Cell growth was monitored turbidimetrically at an optical density of 600 nm (OD_600_). When sugar and sugar acids were concomitantly present in the medium, the concentration of monosaccharide was detected according to the procedures described by Han et al. [44].

## Supplementary Information

**Additional file 1: Fig. S1**. Inhibition of furan aldehydes and phenolics on the growth of *P. putida* KT2440. FAL (a), HMF (b), Vanillin (c), Syringaldehyde (d), 4-Hydroxybenzaldehyde (e), p-Coumaric acid (f), *trans*-Ferulic acid (g). All the experiments were conducted at least in duplicate, and the values were expressed as mean ± standard deviations. **Table S1**. During the detoxification process of engineered *P. putida*, the changes of main fermentable sugars concentrations in 30%(v/v) hydrolysate. **Table S2**. The change of total phenol content in 30%(v/v) hydrolysate with or without detoxified strain. **Table S3**. Plasmids and primers used in construction of the *gcd* and *gtsABCD* knockout strain of *P. putida* KT2440.

## Data Availability

All data generated or analyzed during this study are included in this published article.

## Abbreviations

FAL: Furfural
HMF: 5-Hydroxymethylfurfural
FA: Furoic acid
HMFCA: 5‑Hydroxymethyl‑2‑furancarboxylic acid
*gcd*: Glucose dehydrogenase
*gtsABCD*: Glucose transporter
NaOH: Sodium hydroxide
SSF: Simultaneous saccharification and fermentation
