# Supplementary material for: Efficient lactic acid production from dilute acid-pretreated lignocellulosic biomass by a synthetic consortium of engineered Pseudomonas putida and Bacillus coagulans
Source: Biotechnol Biofuels. 2021 Nov 27;14:227. doi: 10.1186/s13068-021-02078-7 (PMC8627035; doi:10.1186/s13068-021-02078-7)
Supplement: Supplementary file 1 — Additional file 1: Fig. S1. Inhibition of furan aldehydes and phenolics on the growth of P. putida KT2440. FAL (a), HMF (b), Vanillin (c), Syringaldehyde (d), 4-Hydroxybenzaldehyde (e), p-Coumaric acid (f), trans-Ferulic acid (g). All the experiments were conducted at least in duplicate, and the values were expressed as mean ± standard deviations. Table S1. During the detoxification process of engineered P. putida, the changes of main fermentable sugars concentrations in 30%(v/v) hydrolysate. Table S2. The change of total phenol content in 30%(v/v) hydrolysate with or without detoxified strain. Table S3. Plasmids and primers used in construction of the gcd and gtsABCD knockout strain of P. putida KT2440. [file 13068_2021_2078_MOESM1_ESM.docx]

**Electronic Supplementary Information**

**Efficient lactic acid production from dilute acid-pretreated lignocellulosic biomass by** **a** **synthetic** **consortium of engineered *Pseudomonas putida* and *Bacillus coagulans***

Lihua Zou^1^, Shuiping Ouyang^1^, Yueli Hu^1^, Zhaojuan Zheng^1, 2^, Jia Ouyang^1, 2*^

^1^Jiangsu Co-Innovation Center of Efficient Processing and Utilization of Forest Resources, College of Chemical Engineering, Nanjing Forestry University, Nanjing 210037, People’s Republic of China

^2^Jiangsu Province Key Laboratory of Green Biomass-based Fuels and Chemicals, Nanjing 210037, People’s Republic of China

^*^Corresponding author. Address: College of Chemical Engineering, Nanjing Forestry University, Nanjing 210037, People’s Republic of China, Tel.: 86-025-85427129, Fax: 86-025-85427587, E-mail: [hgouyj@njfu.edu.cn](mailto:hgouyj@njfu.edu.cn).

**Materials**

Furan aldehydes and phenolic compounds were purchased from Sigma-Aldrich (USA). All chemicals used in this study were of analytical grade.

**The tolerance analysis of *Pseudomonas putida* KT2440 towards furan aldehydes and phenolics**

To investigate the tolerance of *P. putida* to furan aldehydes and phenolics. Furfural (FAL), 5-hydroxymethylfurfural (HMF) and six typical aromatic compounds were added at different concentrations to the Luria broth (LB) (10 g/L tryptone, 5 g/L yeast extract, and 10 g/L NaCl), respectively. The pH of the medium was adjusted to 7.0 with 5 M sodium hydroxide after addition of the inhibitors. Then, 10% seed culture of *P. putida* was inoculated into medium containing inhibitors, and cultivated at 30 °Cand 200 rpm. The samples were collected periodically for determining the cell growth and the residual substrates.


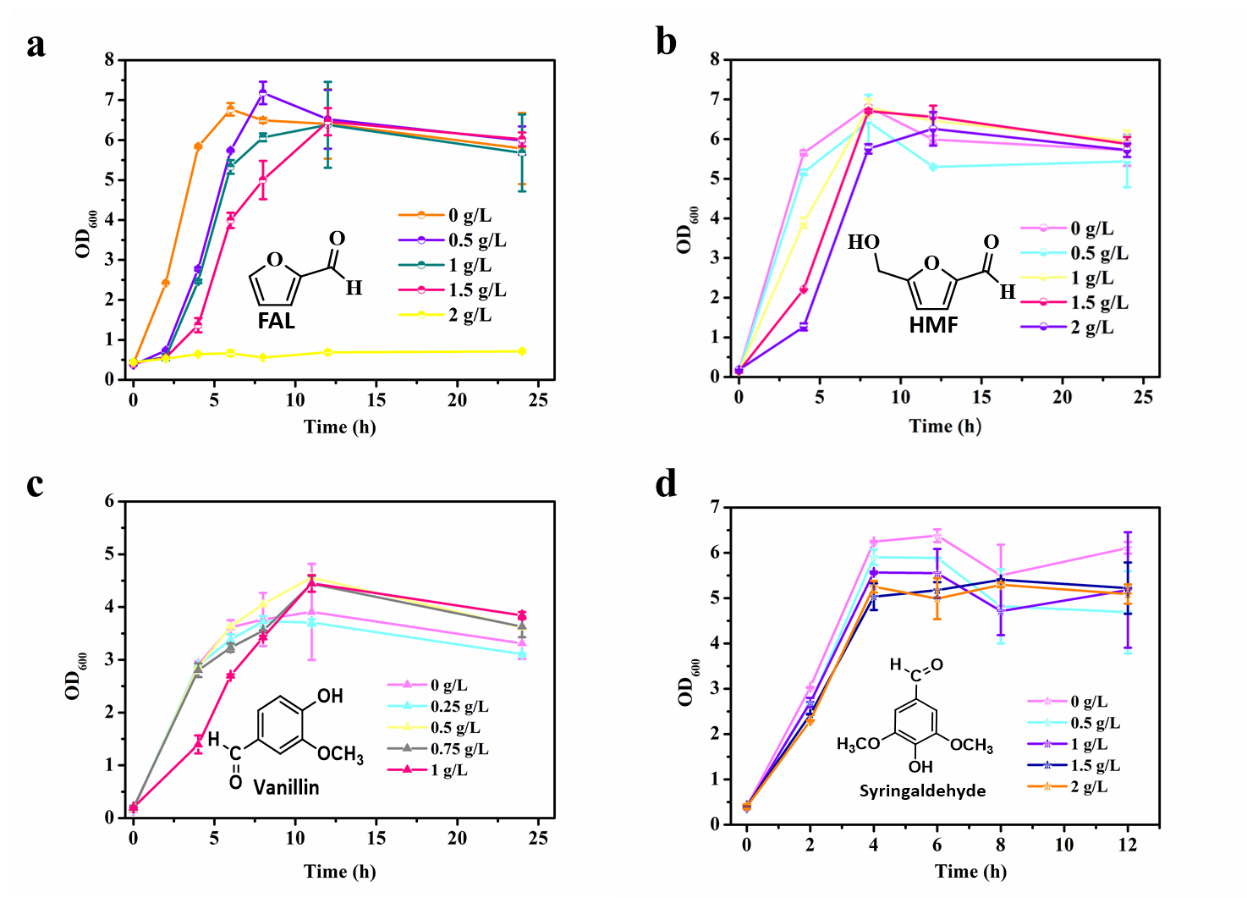


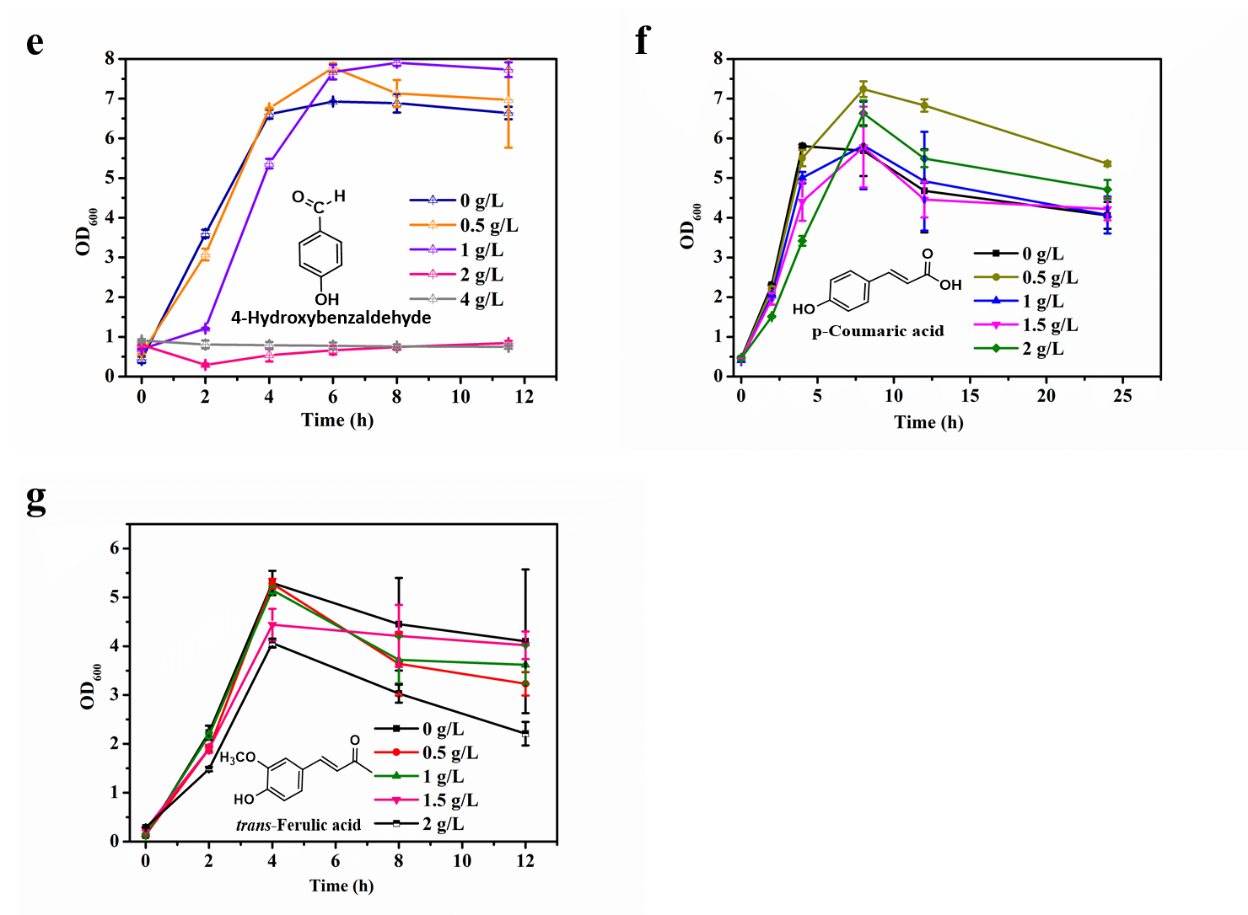


**Fig. S1.** Inhibition of furan aldehydes and phenolics on the growth of *P. putida* KT2440. FAL (a), HMF (b), Vanillin (c), Syringaldehyde (d), 4-Hydroxybenzaldehyde (e), *p*-Coumaric acid (f), *trans*-Ferulic acid (g). All the experiments were conducted at least in duplicate, and the values were expressed as mean ± standard deviations.

**Table S1.** During the detoxification process of engineered *P. putida*, the changes of main fermentable sugars in 30%(v/v) hydrolysate.

| Time  (h) | Concentration (g/L) | | |
| --- | --- | --- | --- |
|  | Glucose | Xylose | Arabinose |
| 0 | 6.7±0.1 | 36.5±0.3 | 4.0±0.0 |
| 12 | 6.5±0.0 | 35.2±0.1 | 4.0±0.0 |
| 24 | 6.5±0.2 | 35.2±0.9 | 4.6±0.9 |

**Table** **S2.** The change of total phenol content in 30%(v/v) hydrolysate with or without detoxified strain.

| Time (h) | Experiment (g/L) | Control (g/L) |
| --- | --- | --- |
| 0 | 1.6±0.0 | 1.6±0.0 |
| 12 | 1.3±0.0 | 1.68±0.0 |
| 24 | 1.2±0.0 | 1.59±0.0 |

Experiment: Addition of detoxifying strains.

Control: No detoxified strain was added.

**Table S3.** Plasmids and primers used in construction of the *gcd* and *gtsABCD* knockout strain of *P. putida* KT2440.

| Name | Relevant characteristic |
| --- | --- |
| Plasmids |  |
| pEASY-Blunt | Cloning vector, Km^r^, Amp^r^ |
| Blunt-Δ*gcd* | pEASY-Blunt containing the fanking regions of *gcd* |
| Blunt-Δ*gtsABCD* | pEASY-Blunt containing the fanking regions of *gtsABCD* |
| pK18mobsacB | Allelic exchange vector, *ori*ColE1 Mob^+^, *lacZα*, *sacB*; Km^r^ |
| pK18MS-Δ*gcd* | The flanking regions of gcd gene were inserted into pK18mobsacB |
| pK18MS-Δ*gtsABCD* | The flanking regions of gtsABCD gene were inserted into pK18mobsacB |
| Primers | Sequence (5’ → 3’) |
| *gcd*-up.f | GGAATTCGCGGCAGTGCCGAGGTGTCGAAGTGGCGGTGG (*Eco*R I) |
| *gcd*-up.r | GGCCTGAAGATCCAGAGCAGTTTCTAACCCGCGACACCGCTCCC GCAGGCTCAACCCTGAGG |
| *gcd*-down.f | GGGTTAGAAACTGCTCTGGATCTTCAGGCC |
| *gcd*-down.r | CGGGATCCGTCAGCCGGCCGCCCTCAGCGGCGCCGCCT (*Bam*H I) |
| *gtsABCD*-up.f | GGAATTCGCATTGTTCGACACAGCCTG (*Eco*R I) |
| *gtsABCD*-up.r | TTATTGATGGTGTAGACGAGCGGAGCACCTTTCTTGTTGT |
| *gtsABCD*-down.f | CTCGTCTACACCATCAATAA |
| *gtsABCD*-down.r | CGGGATCCGTCGAAGTACTTCTGCTTGA (*Bam*H I) |
